# A Randomized, Double-Blind, Placebo-Controlled Trial to Assess the Acidogenic Potential of Dental Biofilms through a Tablet Containing *Weissella cibaria* CMU

**DOI:** 10.3390/ijerph18094674

**Published:** 2021-04-28

**Authors:** Mi-Sun Kang, Dong-Suk Lee, Myoungsuk Kim, Seung-Ah Lee, Seoul-Hee Nam

**Affiliations:** 1 Research and Development Department, Research Institute, OraPharm Inc., Seoul 04782, Korea; jieenkang@orapharm.com; 2 School of Nursing, Kangwon National University, Chuncheon 24341, Korea; ds1119@kangwon.ac.kr (D.-S.L.); cellylife@gmail.com (M.K.); ronjjang@naver.com (S.-A.L.); 3 Department of Dental Hygiene, College of Health Sciences, Kangwon National University, Samcheok 25949, Korea

**Keywords:** *Weissella cibaria*, probiotics, dental biofilms, oral health, clinical study

## Abstract

The possibility of preventing dental caries by taking probiotic bacterium *Weissella cibaria* (*W. cibaria*) CMU tablets to alter the pH of the dental plaque in the oral cavity was evaluated. A randomized, double-blind, placebo-controlled trial was performed on adults aged 20 years or older with 20 or more natural teeth. Ninety-two people underwent dental scaling before being randomly assigned to the experimental group (*n* = 49) or the control group (*n* = 43). Depending on the group they belonged to, *W. cibaria* CMU or the placebo was administered to them once daily for 8 weeks before bedtime. Twenty-four subjects were later excluded from the study because the week 8 dosing was not smoothly performed, for a final subject count of 68. The Cariview test was used to evaluate the amount of acid produced by the dental plaque to assess the risk of caries. The results showed that although there was no significant difference between the results of the two groups, the intake of the *W. cibaria* CMU tablets eliminated the risk of developing dental caries from acid production in the oral flora because the *W. cibaria* colonizes and lives in the dental plaque and the oral cavity and suppresses acids.

## 1. Introduction

With the development of modern medical technology and people’s interest in health, the life expectancy of humans has been extended and the importance of oral health is being recognized. The oral cavity is one of the most populated organs for bacteria. In particular, dental plaque, a biofilm composed of various bacterial colonies, exists on the tooth surface [1]. Subgingival biofilm is a biofilm firmly attached to the tooth above the gum line, and subgingival biofilm is a biofilm firmly attached to the tooth below the gum line. When the homeostasis of the dental plaque is disrupted, the balance of the surrounding environment is also disrupted through a change in the composition of the bacterial community. If this condition persists, it leads to pathological conditions that can cause oral diseases such as dental caries and periodontal disease [2]. Pathogenic bacteria that have the potential to cause dental caries to thrive in acidic environments; those that have the potential to cause periodontal disease to flourish in a slightly alkaline environment.

Dental caries, a chronic disease with the highest morbidity among dental diseases and distributed across a wide range of age groups, is a disease of the hard tissues of the teeth that accounts for most dental treatments [3]. Dental caries occurs when various factors work in combination, among them, disease-causing factors such as microbial factors, host factors, and diet factors [4]. If dental caries is not treated early, it can lead to acute pulpitis that causes severe toothache, which can worsen to chronic pulpitis or pulp necrosis [5].

Since dental caries is caused by organic acids produced by microorganisms, the acidogenic potential of all those microorganisms in plaque is important to evaluate. As various microorganisms interact in a complex and organic manner to form a biofilm on the surface, it is considered important to understand the characteristics of plaque to identify the caries’ activity [6]. The Cariview test measures the level of acid production in the oral biofilm and presents the risk of caries to the subject. By culturing all the microorganisms that produce acid in the plaque, it is possible to evaluate the acidogenic potential of all the bacteria in the plaque. The Cariview test expresses in color, through an indicator, the pH of the organic acids produced by the microorganisms. As the pH decreases, the blue-green -yellow-orange-red color spectrum broadens, and the redder the color is, the higher the risk of dental caries is. That is, this test enables easy and accurate determination of the amount of acid produced in dental plaque in the oral cavity of a patient [7]. Its usefulness in dental caries risk assessment for children and adults in their twenties has been validated [8,9,10]. Recently, attempts to predict the risk of caries by evaluating the activity of all bacteria in the oral cavity have been made for various targets such as children, hematologic tumor patients, and periodontal disease patients [11,12,13].

Probiotics are active microbial supplements that show health-promoting effects by improving the balance of the host’s intestinal microbes [14]. In the current market, the position of probiotics is limited to the intestines; but in recent years, its functionality has been diversifying [15,16,17]. *Weissella cibaria (W. cibaria)* is a gram-positive bacteria species that belongs to the *Leuconostocaceae* family, which is a type of lactobacillus that uses carbohydrates anaerobically to produce lactic acid [18].

According to Kang et al. [19], *W. cibaria* CMU-containing tablets have antimicrobial activity in the oral cavity, as they can selectively control various harmful bacteria that cause periodontal disease, and thus, play an effective role in gum health and show excellence in promoting overall oral health. In addition, according to a study by Lee et al. [20], *W. cibaria* CMU is a safe and useful oral care product for controlling bad breath. However, since the tablets contain *W. cibaria* CMU, which is a lactic acid-producing microorganism, it is necessary to ensure their safety by analyzing the degree of oral acid production according to intake and evaluating the possibility of it inducing dental caries. It is necessary to consider how *W. cibaria* CMU tablets that are taken in a complex oral environment will affect such oral environments. When the pH falls below the 5.5 critical level, at which enamel demineralization begins, the equilibrium between the hydroxyapatite of the enamel and the concentration of calcium and phosphate ions in the plaque covering the enamel surface tilts, causing the enamel to dissolve [21]. Therefore, it is important to maintain the proper acidity (pH) of the teeth to prevent the occurrence of tooth decay. The pH imbalance is considered an important factor in the development of dental caries due to the acids produced by the sugar decomposition from the bacteria in the oral cavity. Therefore, this study evaluated the acidogenic potential of dental plaque through the Cariview test to assess whether there is caries activity after ingestion of the probiotic bacterium *W. cibaria* CMU.

## 2. Materials and Methods

### 2.1. Study Participants

The sample size of the participants was determined based on the medium effect size from the G * Power 3.1 program (Heinrich-Heine-University, Düsseldorf, Germany). The significance level for all the t-tests was set at *p* = 0.05, power = 0.8, and effect size = 0.7. The sample size was planned to be 96 people considering a dropout rate of 40% but, at the beginning of this study, 100 participants volunteered. They were all screened, and 8 subjects were excluded because they did not meet the selection criteria or refused to participate in all three experiment rounds. The inclusion criteria were adults aged 20 years or older who had 20 or more natural teeth that could comply with the protocol, had no tongue problems (tongue cancer and glossitis), or any other inflammation or neoplasia in the oral cavity, and had a concentration of volatile sulfur compounds (VSCs) of 1.5 ng/10 mL or more by Oral Chroma (FIS Inc., Hyogo, Japan) measurement [22]. The exclusion criteria were subjects who had received antibiotic treatment in the last month; those who were receiving dental treatment for severe oral disease at the time of the study; those who had installed a correction device or a fixation device during or after orthodontic treatment; those with adverse reactions to lactose or fermented milk products; those who continued to use probiotic supplements; those with dry mouth; and those with systemic diseases that could cause bad breath. The remaining 92 subjects were randomly assigned to the probiotic test group (*n* = 49) or to the placebo control group (*n* = 43). During the study, 24 more subjects were excluded since the week 8 dosing was not smoothly performed. Thus, the final subject count was 68.

### 2.2. Study Design and Treatments

A randomized double-blind, placebo-controlled trial was performed. For the group assignment of the experimental group and the control group, a research assistant irrelevant to the study used a randomization program (https://www.randomizer.org/, accessed on 28 August 2018) for the assignment to sequentially apply the number of 1 and 0 generated starting from the enrollment number 1. The experimental group was provided *Weissella cibaria* CMU tablets and the control group was provided tablets that had the same shape as the tablets in the experimental group. The tablets in the experimental group were 800-mg tablets that contained 1.0 × 10^8^ colony-forming units (CFU)/g of *W. cibaria*. CMU (oraCMU; OraPharm Inc., Seoul, Korea), besides isomalt, sucralose, peppermint flavor, maltodextrin, and magnesium stearate. They were manufactured by OraPharm Inc. The placebo tablets in the control group were from the same manufacturer and had the same taste, texture, and appearance as the tablets in the other group, except that they did not contain *W. cibaria* CMU. All the subjects were not allowed to use antimicrobial mouthwash and were instructed to take 1 tablet after brushing their teeth before bedtime with the toothbrush and toothpaste that were distributed to them. After the intake, water or food ingestion was not allowed. This procedure was carried out for eight weeks. Then the subjects were asked to visit the dentist for clinical examination in the morning without any oral hygiene practices such as brushing and gargling. Data collection was conducted by two dental hygienists who were trained beforehand to increase the reliability of their measurements under the guidance of a dentist. The data collection was blinded and the examination was performed at the baseline and at week 8, with no distinction between whether the participant was in the experimental group or the placebo group.

### 2.3. Clinical Examination

A week before the experiment was started, to ensure the homogeneity of the oral conditions of the subjects, they were asked to visit M Dental Clinic in Chuncheon, Korea, to undergo an oral examination by a dentist and scaling to ensure that their oral environment would have the same conditions. One week after the scaling, tablet administration was started for both experimental and placebo groups and the baseline acidogenic potential of the dental plaque on the tooth surface of all the subjects was assessed, through the Cariview kit (AIOBIO, Seoul, Korea), based on the manufacturer’s instructions. The Cariview test was then performed eight weeks after the beginning of the tablet intake. During the experiment, all the subjects were provided with the same type of toothpaste and toothbrush and were trained to use them for the entire study period.

The baseline was set as one week after the calculus removal, and the Cariview^TM^ kit (AIOBIO, Seoul, Korea) was used based on the manufacturer’s instructions to evaluate the acidogenic potential of dental plaque on the tooth surface of all the subjects. The Cariview test was performed before and eight weeks after the oral lactobacilli intervention.

### 2.4. Cariview Test

**Figure 1:**
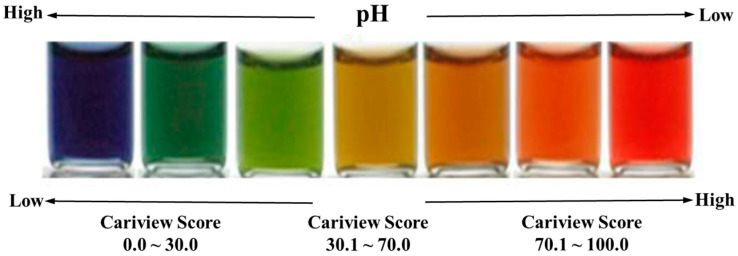
The scales for the Cariview criteria according to the pH value.

On the day of the Cariview test, the subjects were asked not to brush their teeth before their dental visit. After the subjects’ buccal teeth were thoroughly rubbed with a sterilized cotton swab, the cotton swab with plaque was immediately added to the culture solution. The culture solution to which plaque was added was cultured in an incubator at 37 °C for 48 h. The change in the color was observed by dropping 10 drops of the indicator in the kit into the culture solution in which the culture was completed, and the new color was compared with the reference color in the comparison table. Then the new color was photographed with an optical analyzer (Allinone Bio, Seoul, Korea) for a precision test, and the result was scored according to the criteria of the manufacturer for objectivity. As for the Cariview score, the closer to 0 points it was, the lower the acidogenic potential of the dental plaque was and the higher the pH value was; and the closer to 100 points the score was, the higher the acidogenic potential of the dental plaque was and the lower the pH value was. According to the criteria, subjects with a Cariview score of 0.0–40.0 are low-risk; 41.0–70.0, medium-risk; and 71.0–100.0, high-risk (Figure 1).

### 2.5. Ethical Consideration

This study was conducted in accordance with the guidelines of the International Council for Harmonization of Technical Requirements for Pharmaceuticals for Human Use (ICH) and approved by the Kangwon National University (KNU) Institutional Review Board (KWNUIRB-2018-05-003-005, Chuncheon, Korea). The purpose and procedure of the study were explained to all the subjects, and they were further informed that their refusal to participate would not disadvantage them in any way and that they were free to withdraw from the study at any time before they were asked to voluntarily sign the consent form.

### 2.6. Statistical Analysis

An independent *t*-test and Fisher’s exact test were used for confirmation of homogeneity between the two groups at baseline. The normality of the data was confirmed by the Kolmogorov-Smirnov test and the Shapiro-Wilk test. For each variable, depending on the normality of the data, independent *t*-tests or Mann-Whitney tests were performed to compare the absolute values and changes between the two groups before (0 weeks) and after (8 weeks) intake of the tablets. The test results were adjudged as significant when the *p*-value was less than 0.05. In each group, Wilcoxon signed-rank tests were performed to determine whether there was a significant difference in the risk from before to after the intake. All the data were analyzed using SPSS 21.0 for Windows (IBM Corp., Armonk, NY, USA).

## 3. Results

### 3.1. Number of Subjects

**Figure 2:**
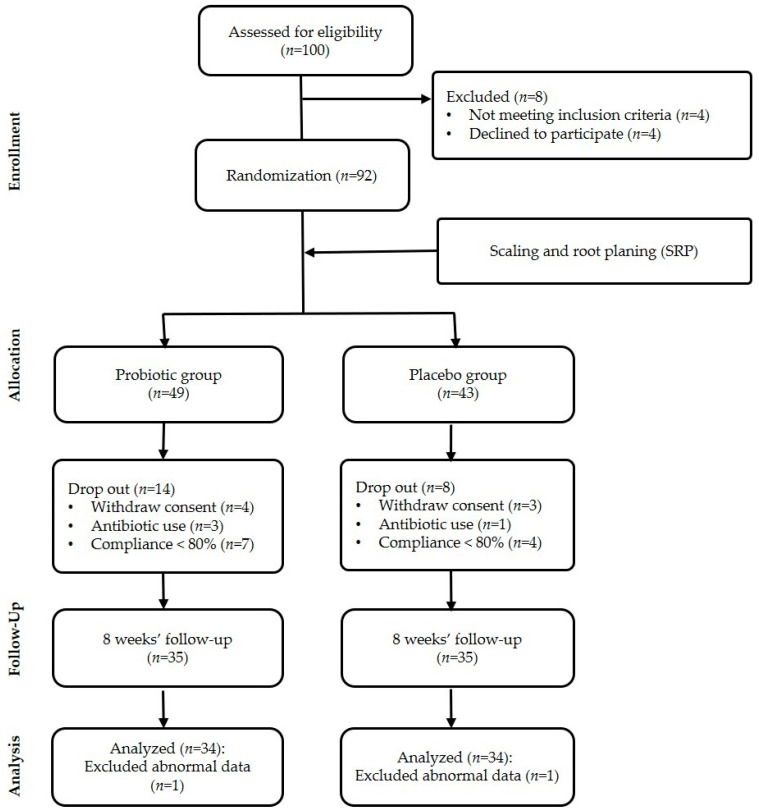
Research flow diagram.

At the start of this study, 100 people who orally agreed to participate in it were recruited. However, during their random assignment to groups and the collection of their written consent, eight people withdrew. Thus, at the baseline (week 0), there were 92 participants, all of whom gave their written consent. Subjects were randomly assigned to the probiotic test group (*n* = 49) or to the placebo control group (*n* = 43). In the middle of the study, seven subjects withdrew due to their inability to make time for the visits, etc., and four subjects dropped out due to their need to take antibiotics for a cold or for wisdom tooth extraction. By the end of the study (week 8), a total of 19 subjects had dropped out, and 81 remained. Of these 81 subjects, 11 were excluded due to less than 80% compliance and two were excluded due to inappropriate data. Thus, 68 subjects remained, and their data were used to analyze the efficacy endpoints (Figure 2).

### 3.2. General Characteristics of the Subjects

Table 1 shows the demographic data and general characteristics of the subjects. The homogeneity of the two groups was confirmed by comparing the general characteristics, including the demographic information, of the subjects. The mean age of the subjects was 23.6 (±3.4) years in the control group and 23.4 (±2.9) years in the study group, showing no significant difference between the two groups (*p* = 0.817). There was also no difference in the distribution of the age group (*p* = 1.000), and in both groups, 20–25 years old was the most common at 85.3%. As for the gender distribution, 15 women and 19 men were in the control group, and 10 women and 24 men were in the study group, showing no significant difference in the χ^2^ test (*p* = 0.209). In addition, there was no significant difference between the two groups in alcohol drinking, smoking, the average number of times they brushed their teeth per day, preliminary oral examination results, and the use of oral hygiene products.

### 3.3. Evaluation of the Dental Caries Activity Index (Cariview)

To compare the dental caries activities, the Cariview score, which predicts the incidence of caries, was evaluated by measuring the acid production rate of oral bacteria. After tablet ingestion for 8 weeks, the Cariview scores showed a statistically significant decrease in both groups (*p* = 0.000), but there was no statistically significant difference between the two groups (Table 2). In addition, the analysis of the distribution according to the risk of caries activity showed that in the *W. cibaria* CMU intake group at eight weeks, the high-risk group decreased from 32.4% to 8.8%, and the low-risk group increased from 0% to 52.9%. In the placebo control group, the high-risk group decreased from 20.6% to 2.9%, and the low-risk group increased from 2.9% to 58.8%. The middle-risk group decreased to 38.2% in both groups, with a statistically significant change in all the analyses (*p* = 0.000) (Table 3). The risk scores were classified into 1 point for the low-risk group, 2 points for the medium-risk group, and 3 points for the high-risk group for analysis between groups according to gender. The results of the statistical analysis along with the Cariview scores and the changes in the Cariview scores at 0 and 8 weeks showed no statistically significant difference between the groups (Table 4).

## 4. Discussion

Dental caries is a disease caused by various factors, so the possibility of developing dental caries cannot be accurately predicted by evaluating only one factor [23,24]. Nevertheless, in the past, the Dentocult system was mainly used as a simple method of determining the risk level of caries; but since only specific strains, *Streptococcus mutans* (*S. mutans*) and *Lactobacilli* sp., were cultivated, there was a limitation in reflecting the various flora related to caries activity in the individual’s oral cavity. In recent years, research using a new Cariview test, which evaluates with a color the acidity of organic acid, the final metabolite secreted by all kinds of microorganisms in plaque, has been actively conducted [7]. The Cariview test is a method of collecting plaque by rubbing the tooth surface with a sterilized cotton swab. Besides the ease with which it obtains a sample, the color change according to the individual’s caries activity level is presented as a score between 0 and 100 points, which more effectively communicates the risk of caries [7].

Based on the ecological plaque hypothesis, it is known that the composition of the microbiota in the dental plaque may change depending on environmental factors, and the risk of caries may also change accordingly [25,26]. Previously, this research team evaluated and reported 16 kinds of periodontal bacteria, including dental caries bacteria, in the four sites of gingival fissures of two maxillary teeth and two mandibular teeth, which had less than 4 mm of periodontal pocket in the *W. cibaria* CMU study group and the placebo control group [19]. In this study, the groups of *Streptococcus* and *Actinomyces viscosus* were statistically significantly higher in the experimental group before tablet intake but showed a statistically significant decrease in the experimental group after 4 weeks of intake. In addition, *Staphylococcus aureus* showed a statistically significant decrease in the experimental group at 8 weeks of intake. *S. mutans*, a major cause of dental caries, showed a greater tendency to decrease in the experimental group, but there was no statistically significant difference between the groups. Most of the other bacteria also showed a higher tendency towards microbial decrease in the experimental group that consumed *W. cibaria* CMU.

On the other hand, in the experimental group that ingested *W. cibaria* CMU, it was confirmed that the number of bacteria increased statistically significantly compared to the control group, as *W. cibaria* settled in the oral cavity [20]. Therefore, in this study, we tried to find out whether there is a risk of caries activity when a tablet containing *W. cibaria*, a lactic acid-producing lactic acid bacteria, is consumed for eight weeks. To improve the oral colonization of *W. cibaria*, it was prepared in the form of tablets that could be sucked in the mouth, and the subjects were instructed to take a tablet in the evening before bedtime after they brushed their teeth. As shown in the study results, the Cariview score in the control group, as well as in the *W. cibaria* intake group, showed a statistically significant decrease compared to before the tablet intake. In addition, in both groups, the risk of dental caries was reduced with the decrease of the middle-risk group and the high-risk group and the increase of the low-risk group. The Cariview score decreased more in the experimental group. Both groups decreased, however, there was no statistical difference between the two groups. In this study, characteristics that could influence the study results were also investigated. Since there was no significant difference between the two groups in age, gender, alcohol drinking, smoking, average number of times the teeth were brushed per day, oral examination, use of oral hygiene products, etc., these variables are considered to have had no effect on the evaluation of the dental caries activity. In addition, there was no significant difference between the groups’ Cariview scores based on their gender and the degree of change.

It has been known that bacteria that produce a lot of lactic acid are not good for oral health because they can cause tooth decay [27]. The higher the PAV (production of acid value) is, the lower the acidogenic potential is [28]. Jang et al. [29] reported that *W. cibaria* CMU had the lowest risk of dental caries because its PAV was higher than that of other oral probiotics, i.e., *Lactobacillus salivarius*, *Lactobacillus reuteri*, and *Streptococcus salivarius* [30]. Moreover, it has been reported from in vitro, in vivo, and clinical studies that *W. cibaria* CMU is a beneficial oral probiotic that improves oral health [31,32,33,34,35]. In particular, in an in vitro study, *S. mutans* converted the process of making water-insoluble glucans using sugar into water-soluble dextran to suppress the formation of biofilm [30]. In beagle dogs as animal models, the plaque index decreased [31]. It was also reported that the plaque index (OHI-S) decreased by 20.7% after ingestion of *W. cibaria* by 72 subjects [30]. In another human study conducted on 60 subjects, *W. cibaria* reduced the plaque index (m-PHP index) with *S. mutans* when ingested for four weeks, and *W. cibaria* was maintained in the oral cavity by 50% compared to before the intake and four weeks after stopping the intake [33]. In this study, Cariview, which can evaluate the acidogenic potential of all microorganisms in the dental plaque, was used to evaluate the caries risk level, and not to measure the number of microorganisms that cause dental caries itself. Although there was no significant difference between the groups, the statistical decrease in the Cariview score was consistent with the previously reported microbial reduction pattern [19].

Oral health practices should be continued to maintain a healthy oral health state prior to the occurrence of oral disease. In this study, the Cariview test was used to determine whether there was a difference in the amount of acid produced by the oral flora to confirm the possibility of tooth demineralization following ingestion of the probiotic bacterium *W. cibaria* CMU. The results of this study confirmed that *W. cibaria* CMU eliminates the risk of dental caries because it reduced the Cariview score, so it is safe to use as an oral probiotic for oral care.

In other studies, the Cariview test has been reported as a more useful caries activity test method in predicting future caries risk and establishing prevention strategies for children. Since this study evaluated dental caries activity in adults over 20 years of age when ingesting tablets containing *W. cibaria* CMU, it seems necessary to evaluate it in children in the future.

## 5. Conclusions

In this study, *W. cibaria* CMU, an oral probiotic, was proven safe to consume since the Cariview test showed that there was no risk of caries activity.

## Figures and Tables

**Table 1:** Baseline characteristics of the subjects in the probiotic and placebo groups (*n* = 68).

Characteristics		Placebo (*n* = 34)	Probiotic (*n* = 34)	χ^2^ or Z or t	*p*-Value
		*n* (%) or M ± SD	*n* (%) or M ± SD		
Age (year)		23.6 ± 3.4	23.4 ± 2.9	−0.230 ^†^	0.817
Age range (year)	20–25	29 (85.3)	29 (85.3)	0.60 ^†^	1.000
	26–30	4 (11.8)	3 (8.8)		
	>31	1 (2.9)	2 (5.9)		
Gender	Female	15 (44.1)	10 (29.4)	1.580	0.209
	Male	19 (55.9)	24 (70.6)		
Drinking	Yes	23 (67.6)	24 (70.6)	0.066	0.793
Smoking	Yes	6 (17.6)	3 (8.8)	1.150 ^†^	0.476
No. of times teeth are brushed per day	None	0 (0.0)	1(2.9)	3.190 ^†^	0.530
	One time	3 (8.8)	1(2.9)		
	Two	10 (29.4)	13 (38.2)		
	Three	14 (41.2)	10 (29.4)		
	Four or more	7 (20.6)	9 (26.5)		
Oral examination	Yes	12 (35.3)	14 (41.2)	0.240	0.618
Oral hygiene	Yes	11 (32.4)	18 (52.9)	2.940	0.086

^†^ Fisher’s exact test; Values are means ± standard deviations or *n* (%).

**Table 2:** Changes in the Cariview scores of the two groups at weeks 0 and 8 (*n* = 68).

Variable	Week	M ± SD			Delta M ± SD		
		Placebo	Probiotic	*p*-Value ^†^	Placebo	Probiotic	*p*-Value ^†^
Cariview	0	61.40±17.53	63.96 ± 19.21	0.722			
	8	43.33±14.58	45.01 ± 16.97	0.589	−18.08 ± 18.25	−18.95 ± 21.37	0.980
	*p*-value ^‡^	0.000	0.000				

^†^ Mann-Whitney test; ^‡^ Wilcoxon signed-rank test; Values are means ± standard deviations.

**Table 3:** Distribution of the subjects according to their risk differences (*n* = 68).

Group	Risk	*n* (%)		*p*-Value ^†^
		0 W	8 W	
Placebo (*n* = 34)	Low	1 (2.9)	20 (58.8)	0.000
	Moderate	26 (76.5)	13 (38.2)	
	High	7 (20.6)	1 (2.9)	
Probiotic (*n* = 34)	Low	0 (0.0)	18 (52.9)	0.000
	Moderate	23 (67.6)	13 (38.2)	
	High	11 (32.4)	3 (8.8)	

^†^ Wilcoxon signed-rank test.

**Table 4:** Comparison of changes in the Cariview and risk scores according to gender (*n* = 68).

	Female (*n* = 25)		Male (*n* = 43)	
	Placebo (*n* = 15)	Probiotic (*n* = 10)	Placebo (*n* = 19)	Probiotic (*n* = 24)
Cariview at 0 W	61.03 ± 18.54	57.45 ± 15.62	61.69 ± 17.21	66.67 ± 20.20
*p*-value ^†^	0.657		0.525	
Cariview at 8 W	41.39 ± 13.09	42.99 ± 12.96	44.85 ± 15.84	45.85 ± 18.58
*p*-value ^†^	0.934		0.493	
Delta Cariview	−16.84	−19.06	−19.64	−14.46
*p*-value ^†^	0.618		0.714	
Risk score at 0 W	2.16 ± 0.50	2.42 ± 0.50	2.20 ± 0.41	2.10 ± 0.32
*p*-value ^†^	0.513		0.112	
Risk score at 8 W	1.53 ± 0.61	1.58 ± 0.72	1.33 ± 0.49	1.50 ± 0.53
*p*-value ^†^	0.414		0.902	

^†^ Mann-Whitney test; Values are means ± standard deviations.

## Data Availability

The data presented in this study are available upon request from the corresponding author. The data are not publicly available due to privacy restrictions.
